# Ultrasonic-Assisted Enzymolysis to Improve the Antioxidant Activities of Peanut (*Arachin conarachin* L.) Antioxidant Hydrolysate

**DOI:** 10.3390/ijms13079051

**Published:** 2012-07-20

**Authors:** Lina Yu, Jie Sun, Shaofang Liu, Jie Bi, Chushu Zhang, Qingli Yang

**Affiliations:** Shandong Peanut Research Institute, Wannianquan Road No. 126, Licang District, Qingdao 266100, China; E-Mails: lhtyln0626@yahoo.com.cn (L.Y.); sj605@sina.com (J.S.); lsf909@sina.com (S.L.); bj.baby@163.com (J.B.); zcs_2003@163.com (C.Z.)

**Keywords:** peanut antioxidant hydrolysate, ultrasonic-assisted enzymolysis, response-surface optimization, antioxidant activities

## Abstract

The objective of this work is to provide a theoretical basis for preparing peanut antioxidant hydrolysate in order to improve its antioxidant activities. Therefore, response surface methodology (RSM) based on the Box-Behnken design was used to optimize ultrasonic-assisted enzymolysis for the purpose of preparing peanut antioxidant hydrolysate. Results indicated that the DPPH free radical scavenging activity of peanut hydrolysate could reach 90.06% under the following optimum conditions: ultrasonic power of 150.0 w, reaction temperature of 62.0 °C, incubation time of 25.0 min, and initial pH value of 8.5. The DPPH free radical scavenging rate of peanut hydrolysate from ultrasonic-assisted enzymolysis improved comparing with that of peanut hydrolysate from protease hydrolysis alone. The peanut antioxidant hydrolysate was found to display eight improved kinds of antioxidant activities. In conclusion, the optimal ultrasonic-assisted enzymolysis technology conditions described in this paper, appear to be beneficial for preparing peanut antioxidant hydrolysate.

## 1. Introduction

Harman proposed the free radical theory in 1956 and believed that the action of free radicals on large biological molecules, leading to histiocyte injury, was the most fundamental reason for aging, tumors, and other diseases in living organisms [1]. In food systems, an excess of free radicals can also seriously influence stability. For example, fat oxidation can change food sensory properties [2]. The products of fat oxidation are also highly toxic in the human body [3]. It is therefore necessary to use antioxidants to scavenge free radicals and control lipid peroxide. Although the synthetic antioxidants butyl hydroxy anisd (BHA) and butylated hydroxytoluene (BHT) have excellent effects, they can injure the liver, spleen, and lungs and they can have cumulative carcinogenic effects [4,5]. Safe, wholesome, and natural antioxidants have received favorable attention [6]. Peptide compounds in long protein chains have been found to inhibit the peroxidation of biological macromolecules and scavenge free radicals. Their antioxidant activities are better than those of proteins or amino acids [7,8]. This type of active peptide is called an antioxidant peptide [9]. In most cases, the main means of preparing antioxidant peptides is to use protease enzymolysis natural animal or plant proteins. The hydrolysate obtained from degreased and roasted peanut kernels using protease hydrolysis has been shown to have good antioxidant activity [10].

Peanut cake is a by-product of the process of making peanut oil. Every year, about three million tons of peanut cakes are produced [11]. The cake is used primarily for feed. However, peanut cake is rich in protein and there are better ways to put this protein resource to use. Using enzymolysis to prepare antioxidant peptides would not only make efficient use of this resource but also expand the scope of finely and deeply processed peanuts, which present remarkable economic and social benefits. Unfortunately, the hydrolysis process has low efficiency, a low final objective product activity, a long reaction time, and higher costs in conventional enzymolysis technology. It is necessary to find a new technique to replace the conventional method.

Ultrasonic-assisted enzymolysis is a relatively new technology [12]. Ultrasonic waves have a frequency greater than 20 kHz [13]. The cavitation, mechanical action, heating effects, and chemical effects are produced during the ultrasonic process [14]. The mechanism of ultrasonic-assisted enzymolysis is as follows [15]. Bubbles obtained from the cavitation burst due to crushing, instantaneously causing enormous mechanical shearing force. Protein degradation and alterations in protein conformation reveal more hydrophilic groups, increasing the protein’s solubility and allowing the protease to more easily bind with the substrate. This increases the efficiency of hydrolysis. At present, no study on ultrasonic-assisted enzymolysis peanut proteins for the purpose of preparing antioxidant hydrolysate has been reported, either domestically or abroad.

Alcalase is a kind of in-depth endo-protease and it can hydrolyze protein to peptides with hydrophobic amino acids at the end of the peptide chain, because it has the high C-terminal hydrophobic amino acids specificity [16]. Peptides prepared from Alcalase hydrolysis have good antioxidant activities. This is because hydrophobic amino acids can intensify the interaction between peptide and fatty acid and they can promote hydrophilic antioxidant amino acid residues to approach lipids. Consequently, the catching capacity for lipid free radicals is improved. In most cases, Alcalase is better suited for hydrolyzing plant protein [17,18].

In general, evaluation methods for antioxidant activities of antioxidant hydrolysate *in vitro* including scavenging free radicals (1,1-diphenyl-2-picrylhydrazyl (DPPH), hydroxyl and superoxide anion free radical), reduction capacity (iron and molybdenum), metal ion chelation (iron and copper ion chelation), anti-lipid peroxidation activity (linoleic acid and lipid peroxidation) [19]. At present, there is no one method to substitute all the standard methods. In view of this, four kinds of antioxidant activities experiments were chosen to comprehensively evaluate antioxidant activities of peanut antioxidant hydrolysate.

In the present study, ultrasonic-assisted Alcalase enzymolysis technology was evaluated and the operational parameters were optimized using single factor-experiments and a response surface methodology (RSM) experimental design in order to increase the amount of antioxidant hydrolysate released and increase its antioxidant activities. At the same time, antioxidant activities of peanut antioxidant hydrolysate were determined. The objective of this work is to provide a theoretical basis for the preparing peanut antioxidant hydrolysate in order to improve its antioxidant activities.

## 2. Results and Discussion

### 2.1. Single-Factor Experiments Results

#### 2.1.1. Effects of Incubation Time with Alcalase on DPPH Free Radical Scavenging Activity of Peanut Hydrolysate

**Figure 1:**
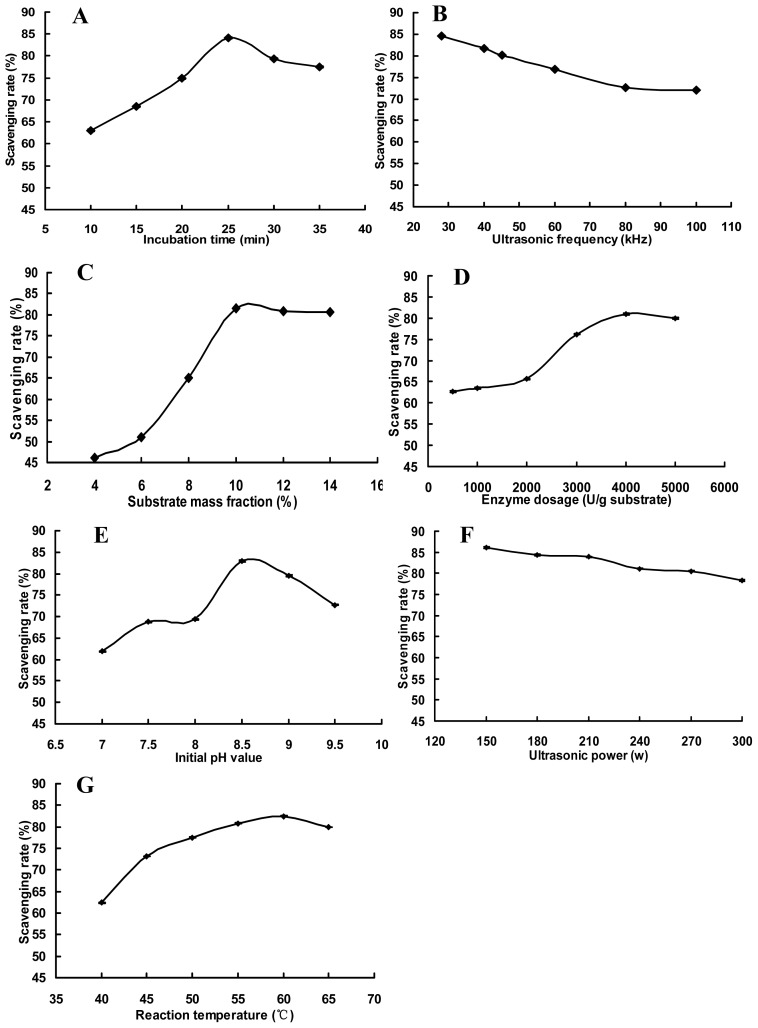
Effects of incubation time (**A**), ultrasonic frequency (**B**), substrate mass fraction (**C**), enzyme dosage (**D**), initial pH value (**E**), ultrasonic power (**F**), reaction temperature (**G**) with Alcalase on 1,1-diphenyl-2-picrylhydrazyl (DPPH) free radical scavenging activity of peanut hydrolysate.

The effects of incubation time on DPPH free radical scavenging activity of peanut hydrolysate are shown in Figure 1A. DPPH free radical scavenging rate first increased, and then decreased. This type of enzymatic reaction is a dynamic equilibrium process. When peanut antioxidant hydrolysate yield is low, the reaction moves from substrate to product. Between 10 and 25 min, DPPH free radical scavenging rate increased, because of generating a small number of peanut antioxidant hydrolysate in a short period of time. Then the enzymatic reaction reached equilibrium (saturation) and the yield of peanut antioxidant hydrolysate reached a peak. The maximum DPPH free radical scavenging rate, then, was observed at an incubation time of 25 min. After 25 min, the peanut antioxidant hydrolysate began to compete with substrate for binding sites and some of them were hydrolyzed into short peptides [20], resulting in both loss of antioxidant activity and decreasing in the DPPH free radical scavenging rate.

#### 2.1.2. Effects of Ultrasonic Frequency with Alcalase on DPPH Free Radical Scavenging Activity of Peanut Hydrolysate

The effects of ultrasonic frequency on DPPH free radical scavenging activity of peanut hydrolysate are shown in Figure 1B. When ultrasonic frequency was increased from 28 kHz to 100 kHz, DPPH free radical scavenging rate decreased first rapidly, and then slowly. Ultrasonic waves are defined as sound waves with frequencies above 20 kHz. When ultrasonic waves travel through the enzymatic reaction solution, there are interactions between the wave, substrate, and protease, producing a series of mechanical, cavitation, heating, and chemical effects. Under low-frequency conditions, mechanical effects will provide shearing action for the substrate protein, loosening its structure. This is beneficial to the enzymatic reaction. As frequency increased, the number of cavitation bubbles increased but the volume of these bubbles became smaller [21]. This generated more numerous and intense shock waves, affecting both substrate and enzyme protein structures, reducing hydrolysis and decreasing DPPH free radical scavenging rate. The ideal ultrasonic frequency was found to be 28 kHz in this experiment.

#### 2.1.3. Effects of Substrate Mass Fraction with Alcalase on DPPH Free Radical Scavenging Activity of Peanut Hydrolysate

The effects of substrate mass fraction on DPPH free radical scavenging activity of peanut hydrolysate are shown in Figure 1C. When the substrate mass fraction was in the range of 4% to 10%, DPPH free radical scavenging rate tended to rise rapidly, and reached a maximum at a 10% mass fraction. When the substrate mass fraction continued to increase, DPPH free radical scavenging rate remained constant. At lower substrate mass fractions, substrate molecules had little chance of binding with proteases, giving a small yield and a low DPPH free radical scavenging rate. As the substrate mass fraction increased, more substrate molecules bound to the active sites of the protease, accelerating the enzymatic reaction from substrate to product. This increased both the yield and the DPPH free radical scavenging rate. However, protease was not enough to hydrolyze substrate when the substrate mass fraction increased to some extent, which resulted in the decreasing of DPPH free radical scavenging rate. The ideal substrate mass fraction was found to be 10% in this paper.

#### 2.1.4. Effects of Enzyme Dosage with Alcalase on DPPH Free Radical Scavenging Activity of Peanut Hydrolysate

Figure 1D illustration shows the effects of enzyme dosage on DPPH free radical scavenging activity of peanut hydrolysate. When the enzyme dosage was in the range of 500–2000 U/g substrate, DPPH free radical scavenging rate increased slowly alongside enzyme dosage. When the enzyme dosage was over 2000 U/g substrate, DPPH free radical scavenging rate became markedly increased, to a maximum at 4000 U/g substrate. As the enzyme dosage continued to increase, DPPH free radical scavenging rate was kept essentially constant. At low enzyme dosages, when the substrate mass fraction was kept constant, many substrate molecules bound to protease active sites to form inactive intermediate products. This caused small yield and low DPPH free radical scavenging rate. When the enzyme dosage increased, proteases bound with more substrate molecules, producing more peanut antioxidant hydrolysate and increasing DPPH free radical scavenging rate. However, the probability of contact between the proteases and substrate molecules decreased when the enzyme dosage increased to some extent, which led to the decreasing of DPPH free radical scavenging rate. The enzyme dosage of 4000 U/g substrate was found to be ideal in this single experiment.

#### 2.1.5. Effects of Initial pH Value with Alcalase on DPPH Free Radical Scavenging Activity of Peanut Hydrolysate

The effects of initial pH value on DPPH free radical scavenging activity of peanut hydrolysate are shown in Figure 1E. DPPH free radical scavenging rate first rose, then declined as initial pH value increased. Results show large DPPH free radical scavenging rates at initial pH 8.5. Under general conditions, pH value can affect protease activity. Proteases are biological catalysts and their catalytic activity is dependent on both spatial structures and active sites [22]. In this experiment, when initial pH value was in the range of 7.0 to 8.5, protease spatial structures changed and more and more active sites became exposed. Then peptide bonds in the substrate protein were broken to generate peanut antioxidant hydrolysate and increase DPPH free radical scavenging rate. When the initial pH value was higher than 8.5, the protease would be denatured due to alkali-modifying effects at high pH. So the production of peanut antioxidant hydrolysate would decrease and DPPH free radical scavenging rate descended.

#### 2.1.6. Effects of Ultrasonic Power with Alcalase on DPPH Free Radical Scavenging Activity of Peanut Hydrolysate

Figure 1F shows the effects of ultrasonic power on DPPH free radical scavenging activity of peanut hydrolysate. As ultrasonic power increased, DPPH free radical scavenging rate decreased. The maximum DPPH free radical scavenging rate was observed at an ultrasonic power of 150 W. The greater the ultrasonic power, the stronger the energy released at a given ultrasonic frequency [16]. The substrate protein molecular structure became loose and peptide bonds became more easily broken to generate peanut antioxidant hydrolysate. However, even greater energy levels produced larger mechanical, cavitation, and heating effects, which can destroy protease molecular conformation, decreasing the efficiency of hydrolysis.

#### 2.1.7. Effects of Reaction Temperature with Alcalase on DPPH Free Radical Scavenging Activity of Peanut Hydrolysate

Figure 1G shows the effects of reaction temperature on DPPH free radical scavenging activity of peanut hydrolysate. DPPH free radical scavenging rate increased as temperature rose over the range of 40 to 60 °C, reaching a maximum at 60 °C. Above 60 °C, DPPH free radical scavenging rate decreased. Reaction temperature is one of the most important elements affecting enzymatic reactions. Rates generally increase as temperature does. This increases the efficiency of hydrolysis, producing more hydrolyzed products and increasing antioxidant activity. When the temperature reaches a certain value, the enzymes and substrates become denatured, decreasing the rate of the reaction.

### 2.2. RSM Experiment Design and Results

#### 2.2.1. Establishment of Model and Analysis of Significance in RSM Experiment Design

According to the Box-Benknken design principle, there are 29 experimental points in an RSM experiment. RSM experimental design and both actual and predicted values of the scavenging rate are given in Table 1. The quadratic regression equation, with dependant variable Y and each factor and its interactions with other the independent variables, was obtained through quadratic multinomial regression and matched the experimental data.

$$\begin{array}{l} Y=80.03-3.08X_{1}+1.23X_{2}-0.15X_{3}+3.87X_{4}+2.85X_{1}X_{2}-1.89X_{1}X_{3}+3.41X_{1}X_{4}-0.48X_{2}X_{3}-\\ 0.65X_{2}X_{4}+1.39X_{3}X_{4}+2.30{X_{1}}^{2}+3.95{X_{2}}^{2}+4.90{X_{3}}^{2}+1.48{X_{4}}^{2} \end{array}$$

The variance analysis of this regression equation is given in Table 2. The regression equation matches experimental data very closely (*p* < 0.001). The regression equation can be used, then, to analyze and predict of actual values of DPPH free radical scavenging rate. The adjusted coefficient of determination (*R*_Adj_^2^), 0.8796, indicates that about 87% of the variation in the scavenging rate is related to the four related factors and that only 13% of the variation remains unaccounted for using this model. The correlation coefficient (*R*), 0.9694, indicates a strong correlation between actual and predicted values. The lack of fit, 0.0691, shows that the experimental results match the response surface model. At the same time, the coefficient of variability (CV) of this model is 2.00%, which also shows the good fit of the regression equation. The design of level intervals of the factors studied is reasonable in term of the Adeq Precision of 15.14.

The significance of the regression coefficients in the quadratic model is given in Table 3. The linear effects of *X*_1_ and *X*_4_ on DPPH free radical scavenging rate are extremely significant (*p* < 0.0001), and that of *X*_2_ is significant (*p* < 0.05). The camber effects of *X*_2_^2^ and *X*_3_^2^ on DPPH free radical scavenging rate are extremely significant (*p* < 0.0001), that of *X*_1_^2^ is highly significant (*p* < 0.01), and that of *X*_4_^2^ is as well significant (*p* < 0.05). The interactions of *X*_1_*X*_2_ and *X*_1_*X*_4_ are highly significant (*p* < 0.01), and that of *X*_1_*X*_3_ is also significant (*p* < 0.05). These results indicate the relationship between these experimental factors and the scavenging rate is not simple or linear. Both quadratic items and interactions have great impact on the dependant value. Comparison of the monomial coefficient of the regression equation, gives us the following list of relevant factors, in order of influence on DPPH free radical scavenging activity of peanut hydrolysate: *X*_4_ > *X*_1_ > *X*_2_ > *X*_3_, or initial pH value > ultrasonic power > reaction temperature > incubation time.

#### 2.2.2. Visual Analysis of RSM Experiment

**Figure 2:**
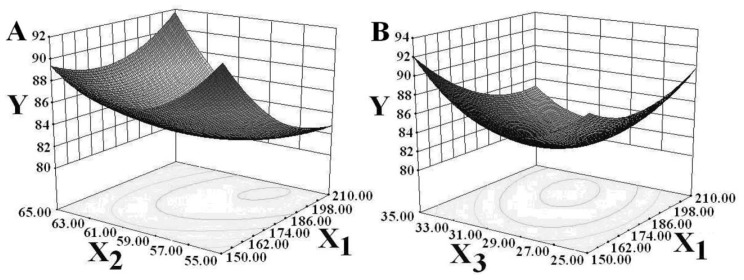

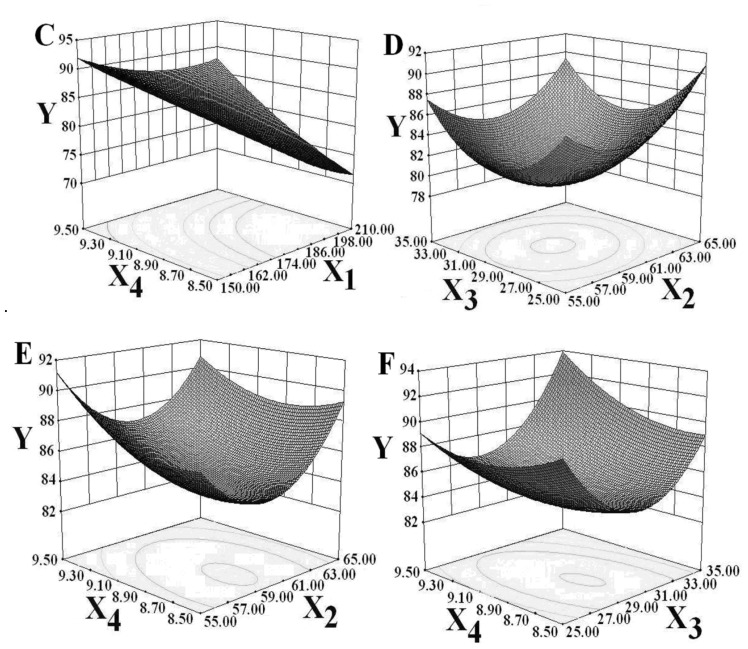
Effects of various factors on scavenging rate of DPPH free radicals (**A**: *X*_1_ and *X*_2_, **B**: *X*_1_ and *X*_3_, **C**: *X*_1_ and *X*_4_, **D**: *X*_2_ and *X*_3_, **E**: *X*_2_, and *X*_4_, **F**: *X*_3_ and *X*_4_; *X*_1_ is ultrasonic power; *X*_2_ is reaction temperature; *X*_3_ is incubation time; *X*_4_ is initial pH value).

By analyzing the RSM regression equation of DPPH free radical scavenging rate, positive correlations were found between the reaction temperature and initial pH value factors and the DPPH free radical scavenging rate. Two other factors, ultrasonic power and incubation time, were found to be negatively correlated with the DPPH free radical scavenging rate. Trend graphs of the interactions between these four factors and the DPPH free radical scavenging rate are given in Figure 2. The influence of the interaction between ultrasonic power and reaction temperature on the scavenging rate is shown in Figure 2A. When the temperature fell below 58 °C, the DPPH free radical scavenging rate showed a rapid decline as ultrasonic power increased. The DPPH free radical scavenging rate increased slowly as ultrasonic power began to increase. In contrast, at reaction temperatures above 58 °C, it rose sharply. At a certain level of ultrasonic power, the DPPH free radical scavenging rate tended to first decrease then increase as temperature rose. The range of change was wider in high temperature. That means the interaction between these two factors was highly statistically significant (*F* = 11.0862, *p* = 0.0050). It can be seen from Figure 2B that the DPPH free radical scavenging rose as ultrasonic power increased when the incubation time was less than 30 min. However, the DPPH free radical scavenging rate decreased significantly when incubation time extended past 30 min. Under given ultrasonic power conditions, the DPPH free radical scavenging rate first decreased and then increased along with time. The interaction between ultrasonic power and incubation time had a somewhat significant effect on the DPPH free radical scavenging rate (*F* = 4.8668, *p* = 0.0446). Figure 2C shows that the DPPH free radical scavenging rate decreased as ultrasonic power increased for fixing initial pH values and increased as initial pH value increased at fixed levels of ultrasonic power. There can then be said to exist a highly statistically significant relationship (*F* = 15.9268, *p* = 0.0013) between ultrasonic power and initial pH value. The DPPH free radical scavenging rate tended to first decrease and then increase, as shown in Figure 2D, 2E, and 2F.

#### 2.2.3. Optimization of Technological Conditions

The optimum conditions for ultrasonic-assisted enzymolysis for the preparation of peanut antioxidant hydrolysate were determined by typical analysis of an experimental model and found to be as follows: the ultrasonic power, 150.07 w; reaction temperature, 62.71 °C; incubation time, 25.01 min; and initial pH value, 8.51. Under the optimum conditions, the predicted value of the DPPH free radical scavenging rate can reach 91.72%. A verification test was conducted in order to confirm the feasibility of RSM. Practical operation factors and convenience were also taken into consideration. The optimum process parameters were modified as follows: the ultrasonic power, 150.0 w; reaction temperature, 62.0 °C; incubation time, 25.0 min; and initial pH value, 8.5. The verification of the test results showed that the DPPH free radical scavenging rate could reach 90.06% ± 0.12 (*n* = 3). The relative error in the difference in the results obtained by prediction and by actual DPPH free radical scavenging rate was less than 2%. This indicates that good fitness between the predicted model and experimental values, confirming the suitability of the prediction model. The DPPH free radical scavenging rate of peanut hydrolysate from Alcalase hydrolysis in water bath is 58.64% ± 0.31 (*n* = 3), which is lower than that of peanut hydrolysate from ultrasonic-assisted enzymolysis by 53.58%. The optimal ultrasonic-assisted enzymolysis technology conditions described in this paper is likely to be suitable for preparing peanut antioxidant hydrolysate under actual-use conditions. It was reported that peanut oligopeptide could be obtained by separation and purification one kind of peanut hydrolysate from Alcalase hydrolyzing peanut protein alone and it had good both antioxidant activities and ACE (angiotensin converting enzyme) inhibitory activity [23]. In conclusion, these results suggest that peanut antioxidant hydrolysate should have better antioxidant activities.

### 2.3. Antioxidant Activities

The four kinds of *in vitro* antioxidant activities including scavenging of free radicals, reduction capacity, metal ion chelation and anti-lipid peroxidation activity of peanut antioxidant hydrolysate prepared by the optimum technological conditions were determined. The results obtained are listed in Table 4. In general conditions, antioxidant mechanism of peanut antioxidant hydrolysate is relevant to types of amino acids (hydrophobic, antioxidant and acidic amino acids), size and sequence of amino acids in peptide chain, molecular weight and spatial conformation [24]. Alcalase, an in-depth endo-enzyme, can hydrolyze peanut protein into peptide with hydrophobic amino acids residue at the end of peptide chain. The hydrophobic amino acids in peptide chain can intensify the interaction between antioxidant peptide and fatty acid, and they can promote hydrophilic antioxidant amino acids residue to approach lipid, so as to improve the catching capacity for lipid free radical [25]. Peanut protein contains a great deal of sequences of amino acids with antioxidant activities. Then antioxidant hydrolysate can be released by using Alcalase under the condition of ultrasonic-assisted enzymolysis. In addition, some amino acids with the supply ability of active hydrogen, such as Trp and Tyr, can form intermediates of free radical, phenoxy free radical and indole free radical, after giving hydrogen atom to free radical. These intermediates can get stable by means of resonance, resulting in the slowing or stopping of free radical chain reaction [26,27].

Of the three scavenging free radical experiments, the scavenging effect on the hydroxyl free radical was the most significant. The hydroxyl free radical is one of the strongest active oxygen species (AOS) that can react with different cell components, such as polysaccharide, amino acids, phospholipids, nucleotide, and organic acids, and it can damage human body to cause aging and disease. Peanut antioxidant hydrolysate showed a powerful ability to scavenge hydroxyl free radical, which indicates that it can be a functional food raw material for protecting health. Free metal ions, such as Fe^2+^ and Cu^+^, can catalyze H_2_O_2_ to be hydroxyl free radical. However, peanut antioxidant hydrolysate containing His has antioxidant effect through chelating transition metal ions. Molecule of His has α-amino group, carboxyl group, and active side chain group imidazolyl. The main chelating mechanism is that α-amino group, carboxyl group and metal ion can give rise to a five ring, α-amino group, imidazolyl and metal ion can form a six ring, and carboxyl group, imidazolyl and metal ion can produce a seven ring. Besides, the interaction of side chain carboxyl group of acidic amino acids in peanut antioxidant hydrolysate and metal ion can passivate metal ion oxidation, and thus weaken free radical chain reaction and achieve antioxidant function. Polyunsaturated fatty acids (PUFA) in lipid, due to containing multiple double bonds, have active chemical properties, and are easy to be destroyed by free radicals and thus set off lipid peroxidation. This peroxidation is one kind of free radical chain reaction. The reaction process is that the hydroperoxide of one unsaturated fatty acid reacts with free radical R·firstly; and then produces double bonds rearrangement reaction; and next forms a 5-membered oxygen ring after absorbing oxygen; and finally, the bond-breaking reaction happens so as to form malonyldiadehyde (MDA). Peanut antioxidant hydrolysate can prevent the reaction between hydroperoxide and free radical R·, and then discontinue auto-oxidation chain reaction to effectively control lipid peroxidation. Reduction capacity is correlated with antioxidant activity. The higher the reduction capacity, the better antioxidant activity of peanut hydrolysate does. Peanut antioxidant hydrolysate can decrease the concentrations of Fe^2+^ and Mu^4+^ in Fenton reaction. The results indicated that peanut antioxidant hydrolysate can prevent oxidative damage and then cardiovascular disease.

## 3. Experimental Section

### 3.1. Chemicals

The low temperature pressed peanut cake was supplied by Shandong Tianshen Biological Protein Co., Ltd. (Linyi, China). Alcalase 2.4 L was purchased from Novozymes A/S. 1,1-diphenyl-2-picrylhydrazyl (DPPH), *O*-phthalic aldehyde (OPA), trichloroacetic acid (TCA), l-α-Phosphatidylcholine, l-Ascorbic acid, thiobarbituric acid (TBA), was purchased from Sigma (St. Louis, MO, USA).

### 3.2. Instruments

Vacuum freezing and drying equipment, Beijing Boyikang Lab Instrument Co., Ltd. (Beijing, China); KQ-300DZ tri-frequency digital full-automatic ultrasonic cleaner, Kunshan Ultrasonic Instruments Co., Ltd. (Shanghai, China); FE20 Laboratory pH Meter, Mettler-toledo Instruments (Shanghai, China) Co., Ltd. (Shanghai, China); High-speed Freezing Centrifuge, Hitachi Koki Co., Ltd (Tokyo, Japan); Ultrospec 2100pro UV-Vis spectrophotometer, Biochrom Co., Ltd (Cambridge, UK).

### 3.3. Peanut Antioxidant Hydrolysate Preparation Technology

Low temperature pressed peanut cake powder was added to petroleum ether and extracted at 50 °C for 2 hours. The defatted peanut cake powder, obtained after drying in ventilation equipment, was added to distilled water to the required mass fraction of suspension and dissolved for 30 min in ultrasonic cleaner. The mixture was adjusted for pH value and added to an Alcalase solution. The enzymatic reaction was carried out in an ultrasonic cleaner at the required temperature for the required length of time. After the reaction was completed, the Alcalase was inactivated at 100 °C for 10 min. The reaction solution was immediately cool to room temperature. Finally, the peanut antioxidant hydrolysate was prepared from the reaction solution, which was treated by centrifuging at 4500 × g for 10 min and settling a certain volume with distilled water.

### 3.4. Experimental Design

The experiment was designed to evaluate seven factors: incubation time, ultrasonic frequency, substrate mass fraction, enzyme dosage, initial pH value, ultrasonic power, reaction temperature, and index of DPPH free radical scavenging activity of peanut hydrolysate.

#### 3.4.1. Single-Factor Experiment

The factors and levels of single-factor experiments are incubation time of 10, 15, 20, 25, 30, 35 min, ultrasonic frequency of 28, 40, 45, 60, 80, 100 kHz, substrate mass fraction of 4.0, 6.0, 8.0, 10.0, 12.0, 14.0%, enzyme dosage of 500, 1000, 2000, 3000, 4000, 5000 U/g substrate, initial pH value of 7.0, 7.5, 8.0, 8.5, 9.0, 9.5, ultrasonic power of 150, 180, 210, 240, 270, 300 W, reaction temperature of 40, 45, 50, 55, 60, 65 °C, respectively. The basic reaction conditions are incubation time of 25 min, ultrasonic frequency of 28 kHz, substrate mass fraction of 10%, enzyme dosage of 4000 U/g substrate, initial pH value of 8.5, ultrasonic power of 150 w and reaction temperature of 60 °C.

#### 3.4.2. Response Surface Methodology, RSM

On the base of single-factor experiments, by the use of a Box-Benhnken design, the four factors (including ultrasonic power (*X*_1_), reaction temperature (*X*_2_), incubation time (*X*_3_), and initial pH value (*X*_4_)) that had significant influences on DPPH free radical scavenging activity of peanut hydrolysate were selected for a RSM experiment involving four factors and three levels at a starting fixed ultrasonic frequency of 28 kHz, substrate mass fraction of 10%, and enzyme dosage of 4000 U/g substrate. The levels of four factors are ultrasonic power of 150, 180, 210 W, reaction temperature of 55, 60, 65 °C, incubation time of 25, 30, 35 min, initial pH value of 8.5, 9.0, 9.5, respectively. The DPPH free radical scavenging rate (*Y*) was taken the dependent variable. Experimental results (Table 1) were analyzed using Design Expert software (Version 8.0; Static Made Easy: Minneapolis, MN, USA, 2011).

### 3.5. Methods

#### 3.5.1. DPPH Free Radical Scavenging Activity

DPPH free radical scavenging activity was determined according to the method of Khantaphant *et al*., [28]. Three kinds of reaction solution, (1) 2 mL of sample solution and 2 mL of 4 mmol/L DPPH, (2) 2 mL of sample solution and 2 mL of anhydrous ethyl alcohol, and (3) 2 mL of 4 mmol/L DPPH and 2 mL of distilled water, were placed in three test tubes and mixed vigorously. After incubation at 25 °C in a water bath for 20 min, the absorbances of the resulting solutions were recorded at 517 nm using a spectrophotometer. The absorption values of tubes 1, 2, and 3 were *A_i_*, *A_j_*, and *A_0_*, respectively. The DPPH free radical scavenging rate can be calculated as follows:

(1) $$\text{DPPH free radical scavenging rate }(\%)=(1-\frac{A_{i}-A_{j}}{A_{0}})\times 100$$

#### 3.5.2. Hydroxyl Free Radical Scavenging Activity

Hydroxyl free radical scavenging activity was determined according to the method of Amarowicz *et al*., [29]. 2 mL of sample solution, 2 mL of sample solution, and 2 mL of distilled water were placed in three test tubes, respectively. Then 2 mL of FeSO_4_ (6 mmol/L) and H_2_O_2_ (6 mmol/L) were added to above three tubes, respectively and mixed vigorously. After standing for 10 min at room temperature, three 2 mL of solutions salicylic acid (6 mmol/L), distilled water and salicylic acid (6 mmol/L) were added to the above three tubes, respectively. After standing for 30 min, the absorbances of the resulting solutions were recorded at 510 nm using a spectrophotometer. The absorption values of tubes 1, 2, and 3 were *A_i_*, *A_j_*, and *A_0_*, respectively. The hydroxyl free radical scavenging rate can be calculated as follows:

(2) $$\text{Hydroxyl free radical scavenging rate }(\%)=(1-\frac{A_{i}-A_{j}}{A_{0}})\times 100$$

#### 3.5.3. Superoxide Anion Free Radical Scavenging Activity

Superoxide anion free radical scavenging activity was determined according to the method of Yu *et al*., [30]. 2 mL of sample solution, 2 mL of sample solution, and 2 mL of distilled water were placed in three test tubes, respectively. Then 2 mL of ammonium persulfate (1%), TEMED (0.1%) and oxammonium hydrochloride (0.1%) were added to three tubes, respectively and mixed vigorously. After incubation at 25 °C in a water bath for 60 min, three kinds of solutions, (1) 1 mL of *p*-aminobenzene sulfonic acid (0.33%) and α-naphthyl amine (1%), (2) 1 mL of *p*-aminobenzene sulfonic acid (0.33%) and distilled water, and (3) 1 mL of p-aminobenzene sulfonic acid (0.33%) and α-naphthyl amine (1%), were added to above tubes, respectively. After standing for 20 min, the absorbances of the resulting solutions were recorded at 530 nm using a spectrophotometer. The absorption values of tubes 1, 2, and 3 were *A_i_*, *A_j_*, and *A_0_*, respectively. The superoxide anion free radical scavenging rate can be calculated as follows:

(3) $$\text{Superoxide anion}\;\text{free radical scavenging rate }(\%)=(1-\frac{A_{i}-A_{j}}{A_{0}})\times 100$$

#### 3.5.4. Iron Reduction Capacity

Iron reduction capacity was determined according to the method of You *et al*., [31]. 1.0 mL of sample, 2.5 mL of phosphate buffer solution (pH 6.6, 0.1 mol/L) and 2.5 mL K_3_Fe(CN)_6_ (1%) were placed in test tube and mixed vigorously. After incubation at 50 °C in a water bath for 20 min, 2.5 mL of TCA (10%) was added to the mixture and centrifuged for 10 min at 3000× g. Then 2.5 mL of supernatant was added to 2.5 mL of distilled water and 0.5 mL FeCl_3_ (0.1%) and left to stand for 10 min after uniform mixing. Finally, the effect of iron reduction capacity was determined by the absorbance value at 700 nm.

#### 3.5.5. Molybdenum Reduction Capacity

Molybdenum reduction capacity was determined according to the method of Yu *et al*., [32]. 1.0 mL of sample was added to 4.0 mL of phosphorus molybdenum blue reagent. After incubation at 95 °C in a water bath for 90 min, the effect of molybdenum reduction capacity was determined by the absorbance value at 695 nm.

#### 3.5.6. Iron Ion Chelation

Iron ion chelation was determined according to the method of Wang *et al*., [33]. 1 mL of sample, 2.7 mL of distilled water and 0.1 mL of FeCl_2_ (2 mmol/L) were placed in test tube and mixed vigorously. The mixture was then reacted with 0.2 mL of ferrozine (5 mmol/L) for 10 min. The control was conducted in the same manner, except that distilled water was used instead of sample. The absorbances of sample (*A*) and control (*A_0_*) were recorded at 562 nm using a spectrophotometer. Iron ion chelating rate was calculated as follows:

(4) $$\text{Iron ion chelaing rate}=(1-\frac{A}{A_{0}})*100\%$$

#### 3.5.7. Copper Ion Chelation

Copper ion chelation was determined according to the method of You *et al*., [34]. 1 mL of sample, 1.0 mL of CuSO_4_ (2 mmol/L), 1.0 mL of pyridine (pH 7.0) and 20 μL of pyrocatechol violet (0.1%) were mixed and left to stand for 5 min. The control was conducted in the same manner, except that distilled water was used instead of sample. The absorbances of sample (*A*) and control (*A_0_*) were recorded at 632 nm using a spectrophotometer. Copper ion chelating rate was calculated as follows:

(5) $$\text{Copper ion chelaing rate}=(1-\frac{A}{A_{0}})*100\%$$

#### 3.5.8. Anti-Lipid Peroxidation Activity

Anti-lipid peroxidation activity was determined according to the method of Yi *et al*., [35]. 1 mL of sample solution, 1 mL of l-α-Phosphatidylcholine, 1 mL of FeCl_3_ (0.4 mmol/L) and 1 mL of l-Ascorbic acid (0.4 mmol/L) were mixed and incubated at 37 °C in a water bath for 60 min in dark. Then, the mixture was added to 2 mL of TCA-TBA-HC1 and incubated at 95 °C in a water bath for 15 min. The control was prepared in the same method except that distilled water was used instead of sample. The absorbances of sample (*A*) and control (*A_0_*) were recorded at 535 nm using a spectrophotometer. Inhibition rate of anti-lipid peroxidation activity was calculated as follows:

(6) $$\text{Inhibition rate}=(1-\frac{A}{A_{0}})*100\%$$

### 3.6. Statistical Analysis

All experiments were performed in triplicate and each data point was expressed as the mean and standard deviation. The significance level of *p* < 0.05 was employed.

## 4. Conclusions

Based on single-factor experiments, RSM design was applied to ultrasonic-assisted enzymolysis in the preparation of peanut antioxidant hydrolysate. Comparing the ultrasonic-assisted enzymolysis with Alcalase hydrolysis, the former required less time and had higher antioxidant activities. From these results, it appears that ultrasonic-assisted enzymolysis may be used to obtain good antioxidant hydrolysate from peanut cake with potential application in food products to prevent lipid oxidation or to increase nutritional values. Further research will focus on the purification, amino acid sequence analysis, recombinant DNA techniques synthesis, antioxidant mechanism, and structure–activity relationship studies of peanut antioxidant peptide by using this peanut antioxidant hydrolysate.

## Figures and Tables

**Table 1:** Response surface methodology (RSM) experiment design and results.

Number	*X*_1_ (w)	*X*_2_ (°C)	*X*_3_ (min)	*X*_4_	*Y* (actual values) (%)	*Y*’ (predicted values) (%)
1	150	55	30	9	90.22	90.98
2	210	55	30	9	80.94	19.12
3	150	65	30	9	87.73	87.74
4	210	65	30	9	89.83	87.28
5	180	60	25	8.5	86.53	84.08
6	180	60	35	8.5	82.61	81.00
7	180	60	25	9.5	89.23	89.04
8	180	60	35	9.5	90.87	91.52
9	150	60	30	8.5	86.57	86.43
10	210	60	30	8.5	72.65	73.45
11	150	60	30	9.5	88.35	87.35
12	210	60	30	9.5	88.07	88.01
13	180	55	25	9	86.51	87.32
14	180	65	25	9	90.46	90.74
15	180	55	35	9	88.47	87.98
16	180	65	35	9	90.50	89.48
17	150	60	25	9	88.62	88.57
18	210	60	25	9	84.58	86.19
19	150	60	35	9	91.64	92.05
20	210	60	35	9	80.06	82.11
21	180	55	30	8.5	78.65	79.71
22	180	65	30	8.5	81.11	83.47
23	180	55	30	9.5	89.08	88.75
24	180	65	30	9.5	88.95	89.91
25	180	60	30	9	79.36	80.03
26	180	60	30	9	81.19	80.03
27	180	60	30	9	80.11	80.03
28	180	60	30	9	79.00	80.03
29	180	60	30	9	80.49	80.03

**Table 2:** Variance analysis of regression model.

Source	Sum of squares	df	Mean square	*F* value	*p*-value Prob > *F*
Model	638.2259	14	45.5876	15.6101	<0.0001
Residual	40.8854	14	2.9204	/	/
Lack of fit	37.8120	10	3.7812	4.9212	0.0691
Pure error	3.0734	4	0.7684	/	/
Cor total	679.1113	28	/	/	/

*R*^2^ = 0.9398; *R*_Adj_^2^ = 0.8796; CV(%) = 2.0045; *R*_Pred_^2^ = 0.6722; Adeq Precision = 15.1409

**Table 3:** Significance test for regression coefficient.

Factor	Coefficient estimate	df	Standard error	95% CI low	95% CI high	*F* value	*p*-value Prob > *F*
Intercept	80.0300	1	0.7643	78.3909	81.6692	15.6101	<0.0001
*X*^1^	−3.0833	1	0.4933	−4.1414	−2.0253	39.0645	<0.0001
*X*^2^	1.2258	1	0.4933	0.1678	2.2839	6.1745	0.0262
*X*^3^	−0.1483	1	0.4933	−1.2064	0.9097	0.0904	0.7681
*X*^4^	3.8692	1	0.4933	2.8111	4.9272	61.5142	<0.0001
*X*^1^*X*^2^	2.8450	1	0.8545	1.0124	4.6776	11.0862	0.0050
*X*^1^*X*^3^	−1.8850	1	0.8545	−3.7176	−0.0524	4.8668	0.0446
*X*^1^*X*^4^	3.4100	1	0.8545	1.5774	5.2426	15.9268	0.0013
*X*^2^*X*^3^	−0.4800	1	0.8545	−2.3126	1.3526	0.3156	0.5832
*X*^2^*X*^4^	−0.6475	1	0.8545	−2.4801	1.1851	0.5742	0.4611
*X*^3^*X*^4^	1.3900	1	0.8545	−0.4426	3.2226	2.6464	0.1261
*X*_1_^2^	2.2996	1	0.6710	0.8605	3.7387	11.7454	0.0041
*X*_2_^2^	3.9483	1	0.6710	2.5092	5.3875	34.6256	<0.0001
*X*_3_^2^	4.9020	1	0.6710	3.4630	6.3412	53.3741	<0.0001
*X*_4_^2^	1.4758	1	0.6710	0.0367	2.9150	4.8378	0.0451

Factors found to have significant effects on the scavenging rate of DPPH free radicals (*p* ≤ 0.05); factors found to have highly significant effects on the scavenging rate of DPPH free radicals (*p* ≤ 0.01); factors found to have extremely significant effects on the scavenging rate of DPPH free radicals (*p* ≤ 0.001); factors found to have no significant effect on the scavenging rate of DPPH free radicals (*p* > 0.05).

**Table 4:** Results of antioxidant activities.

	Regression Equation	*R*^2^	IC_50_ (mg/mL)
DPPH free radical scavenging activity	y = −0.1862x^2^ + 9.2691x + 2.6819	0.9999	5.77
Hydroxyl free radical scavenging activity	y = −0.00005x^2^ + 0.09694x + 17.56407	0.9951	0.43
Superoxide anion free radical scavenging activity	y = −0.167x^2^ + 7.8646x + 2.0721	0.9991	7.19
Iron ion chelation	y = −0.0627x^2^ + 6.9982x + 0.4071	0.9986	7.60
Copper ion chelation	y = −0.0476x^2^ + 5.0688x − 1.0542	0.9999	11.26
Anti-lipid peroxidation activity	y = −0.1025x^2^ + 7.2155x + 0.2006	0.9986	7.76
Iron reduction capacity	y = 0.0387x + 0.0097	0.9997	12.67
Molybdenum reduction capacity	y = 0.0991x + 0.0592	0.9994	4.45

## References

[1] Harman D. (1956). Aging: A theory based on free radical and radiation chemistry. J. Gerontol.

[2] Pastorelli G., Magni S., Rossi R., Pagliarini E., Baldini P., Dirinck P., van Opstaele F., Corino C. (2003). Influence of dietary fat, on fatty acid composition and sensory properties of dry-cured Parma ham. Meat Sci.

[3] Ren J., Zheng X.Q., Liu X.L., Liu H. (2010). Purification and characterization of antioxidant peptide from sunflower protein hydrolysate. Food Technol. Biotechnol.

[4] Rajaram D., Nazeer R.A. (2010). Antioxidant properties of protein hydrolysates obtained from marine fishes Lepturacanthus savala and Sphyraena barracuda. Int. J. Biotechnol. Biochem.

[5] Sheih I.C., Wu T.K., Fang T.J. (2009). Antioxidant properties of a new antioxidative peptide from algae protein waste hydrolysate in different oxidation systems. Bioresour. Technol.

[6] Radwan N.L., Hassan R.A., Qota E.M., Fayek H.M. (2008). Effect of natural antioxidant on oxidative stability of eggs and productive and reproductive performance of laying hens. Int. J. Poult. Sci.

[7] Sheih I.C., Fang T.J., Wu T.K., Lin P.H. (2010). Anticancer and antioxidant activities of the peptide fraction from algae protein waste. J. Agric. Food Chem.

[8] Gao D.D., Cao Y.S., Li H.X. (2010). Antioxidant activity of peptide fractions derived from cottonseed protein hydrolysate. J. Sci. Food Agric.

[9] Liu B.L., Chiang P.S. (2008). Production of hydrolysate with antioxidative activity and functional properties by enzymatic hydrolysis of defatted sesame (*Sesamum indicum* L.). Int. J. Appl. Sci. Eng.

[10] Hwang J.Y., Shue Y.S., Chang H.M. (2001). Antioxidative activity of roasted and defatted peanut kernels. Food Res. Int.

[11] Girma M., Tamir B., Dessie T. (2012). Effects of replacing peanut seed cake with brewery dried yeast on laying performance, egg quality and carcass characteristics of rhode island red chicken. Int. J. Poult. Sci.

[12] Karaboğa C., Körlü A.E., Duran K., Bahtiyari M.İ (2007). Use of ultrasonic technology in enzymatic pretreatment processes of cotton fabrics. Fibres Text. East. Eur.

[13] Dolatowski Z.J., Stadnik J., Stasiak D. (2007). Applications of ultrasound in food technology. Acta Sci. Pol. Hortoru.

[14] Fernandes F.A.N., Gallão M.I., Rodrigues S. (2008). Effect of osmotic dehydration and ultrasound pre-treatment on cell structure: Melon dehydration. LWT Food Sci. Technol.

[15] Patist A., Bates D. (2008). Ultrasonic innovations in the food industry: From the laboratory to commercial production. Innov. Food Sci. Emerg.

[16] Muthukumaran S., Kentish S.E., Ashokkumar M., Stevens G.W. (2005). Mechanisms for the ultrasonic enhancement of dairy whey ultrafiltration. J. Membr. Sci.

[17] Vercruysse L., Smagghe G., Beckers T., Camp J.V. (2009). Antioxidative and ACE inhibitory activities in enzymatic hydrolysates of the cotton leafworm, Spodoptera littoralis. Food Sci.

[18] Zhang J.H., Zhang H., Wang L., Guo X.N., Wang X.G., Yao H.Y. (2010). Isolation and identification of antioxidative peptides from rice endosperm protein enzymatic hydrolysate by consecutive chromatography and MALDI-TOF/TOF MS/MS. Food Chem.

[19] Decker E.A., Warner K., Richards M.P., Shaidi F. (2005). Measuring antioxidant effectiveness in food. J. Agric. Food Chem.

[20] Sorokin A.V., Kim E.R., Ovchinnikov L.P. (2009). Proteasome system of protein degradation and processing. Biochemistry (Moscow).

[21] Sangave P.C., Pandit A.B. (2006). Ultrasound and enzyme assisted biodegradation of distillery wastewater. J. Environ. Manag.

[22] Veselovsky A.V., Medvedev A.E., Tikhonova O.V., Skvortsov V.S., Ivanov A.S. (2000). Modeling of substrate-binding region of the active site of monoamine oxidase A. Biochemistry (Moscow).

[23] Zhang Y.H., Wang Q. (2007). Peanut protein hydrolyzing by Alcalase to prepare peanut oligopeptides. Trans. CSAE.

[24] Dávalos A., Miguel M., Bartolomé B., López-Fandiño R. (2004). Antioxidant activity of peptides derived from egg white proteins by Enzymatic Hydrolysis. J. Food Prot.

[25] Lee W.S., Jeon J.K., Byun H.G. (2011). Characterization of a novel antioxidative peptide from the sand eel Hypoptychus dybowskii. Process Biochem.

[26] Rajapakse N., Mendis E., Byun H.G., Kim S.K. (2005). Purification and *in vitro* antioxidative effects of giant squid muscle peptides on free radical-mediated oxidative systems. J. Nutr. Biochem.

[27] Hsu K.C. (2010). Purification of antioxidative peptides prepared from enzymatic hydrolysates of tuna dark muscle by-product. Food Chem.

[28] Khantaphant S., Benjakul S., Ghomi M.R. (2011). The effects of pretreatments on antioxidative activities of protein hydrolysate from the muscle of brownstripe red snapper (*Lutjanus vitta*). LWT Food Sci. Technol.

[29] Amarowicz R., Naczk M., Shahidi F. (2000). Antioxidant activity of various fractions of non-tanin phenolics of canola hulls. J. Agric. Food Chem.

[30] Yu L.N., Gong Q.X., Yang Q.L., Sun J., Bi J., Zhang C.S. (2011). Technology optimization on microwave-assisted extraction water soluble dietary fiber from peanut hull and its antioxidant activity. Food Sci. Technol. Res.

[31] You L.J., Zhao M.M., Cui C., Zhao H.F., Yang B. (2009). Effect of degree of hydrolysis on the antioxidant activity of loach (*Misgurnus anguillicaudatus*) protein hydrolysates. Innov. Food Sci. Emerg.

[32] Yu L.N., Yang Q.L., Yu S.L., Bi J., Zhang C.S. (2009). Study On the Extraction of Water Soluble Dietary Fiber and Antioxidant Activity from Peanut Stem by Ultrasonic Wave.

[33] Wang X.S., Tang C.H., Chen L., Yang X.Q. (2009). Characterization and antioxidant properties of hemp protein hydrolysates obtained with neutrase. Food Technol. Biotechnol.

[34] You L.J., Zhao M.M., Regenstein J.M., Ren J.Y. (2011). *In vitro* antioxidant activity and *in vivo* anti-fatigue effect of loach (*Misgurnus anguillicaudatus*) peptides prepared by papain digestion. Food Chem.

[35] Asha V.V., Akhila S., Wills P.J., Subramoniam A. (2004). Further studies on the antihepatotoxic activity of *Phyllanthus maderaspatensis* Linn. J. Ethnopharmacol.

